# Effects of Induced Moisture Loss in Chicken Embryos at Embryonic Day 18 and Post-hatch Immune Response During *Salmonella enteritidis* Lipopolysaccharide Challenge in Broilers

**DOI:** 10.3389/fphys.2022.820349

**Published:** 2022-03-07

**Authors:** Jenna L. Gregorich, Michael S. Lilburn, Revathi Shanmugasundaram

**Affiliations:** ^1^Department of Animal Sciences, The Ohio State University, Wooster, OH, United States; ^2^U.S National Poultry Research Center, Athens, GA, United States

**Keywords:** chicken embryos, induced moisture loss, inflammatory challenge, CD8^+^/CD4^+^ ratio, Tregs percentage, cytokine response

## Abstract

Two experiments were conducted to investigate the effects of induced moisture loss on embryonic development and the immune response following an inflammatory challenge immediately post-hatch. In Experiment I, fertile leghorn eggs (*n* = 100) and commercial broiler eggs (*n* = 300) were set at 37.5°C and moisture loss was induced in one-half of the Leghorn and broiler eggs by drilling two, 1.5 mm diameter holes. The Control eggs had 0 holes. At embryonic day (ED)18, layer and broiler eggs in the 2-holes treatment had a significant (*P* < 0.01) increase in moisture loss compared to the control treatment (10.1% vs. 8.2%). Similarly, at ED18, the broiler eggs with 2-holes had a significant increase (*P* < 0.01) in moisture loss compared with control eggs (9.9% vs. 8.4%). Thymocytes from both the leghorn (104%) and broiler (62%) embryos in the 2-holes treatment had significantly increased *in vitro* proliferation compared with the control embryos (*P* ≤ 0.05). At ED18, layer and broiler embryos in the 2-holes treatment had an approximate twofold increase in the splenic CD8^+^/CD4^+^ ratio (*P* ≤ 0.05) and CD4^+^CD25^+^ cells percentage in both the thymus and spleen (*P* ≤ 0.05). At ED18, both layer and broiler embryos from the 2-holes treatment had a significant increase in splenic IL1-β, IL-6, IL-10, and TLR-4 mRNA transcription compared to the control group (*P* ≤ 0.05). Experiment II was repeated with 300 fertile broiler eggs. On the day of hatch, chicks were randomly distributed into one of four treatments in a 2 (0, 2 holes) × 2 (0, 500 μg lipopolysaccharide, LPS) factorial arrangement of treatments. Chicks in the LPS groups were injected intraperitoneally with 500 μg/kg BW LPS. At 24 and 48 h post-hatch, chicks hatched from eggs with 2-holes and challenged with LPS had a significant increase (*P* ≤ 0.05) in thymocyte proliferation at 24 h (42%) and 48 h (37%) when compared with chicks hatched from the control (0-hole; 0 μg LPS) treatment. Chicks hatched from the 2-holes treatment and challenged with the LPS had an approximately twofold higher splenic CD8^+^/CD4^+^ ratio and 1.5 fold increase in CD4^+^CD25^+^ percentage compared to control chicks (P ≤ 0.05). In chicks hatched from the 2-holes treatment, MUC2 mRNA transcription was comparable to control chicks at 24 and 48 h in response to the LPS challenge. Our data suggest that the 2-holes treatment reprograms gene transcription to facilitate cell survival via proliferation and differentiation during an LPS inflammatory challenge.

## Introduction

For successful hatching, developing avian embryos require an ideal temperature, turning, humidity, and ventilation (Romanoff, 1929). Avian eggs lose water during incubation and monitoring moisture loss during incubation is critical to achieving optimal hatchability and chick quality (Hoyt, 1979). Moisture loss via diffusion through eggshell pores occurs steadily during incubation and is greater than the metabolic water produced by embryonic oxidation of yolk lipids (Ar and Rahn, 1980). Incubator humidity is one of the important factors in controlling water loss during incubation (Landauer, 1967). The moisture loss percentage can range from 10 to 13% in multi-stage incubators and 9.5–12.5% of initial egg mass in single-stage incubators (Green, 2017). Experimentally, induced moisture loss (>20%) achieved by drilling holes in the eggshell over the airspace on an embryonic day (ED)1 caused osmotic stress due to early depletion of allantoic fluid (Davis, 1987).

Changes in the osmotic state can negatively influence an organism’s function and the capacity of avian embryos to regulate hydration is critical for optimal hatchability (Moeller et al., 2013). Natural variation in the number of pores in an eggshell between individual eggs within a single population contributes to the variability in moisture loss during incubation and this variability can subsequently influence osmoregulation by the embryo (Tullett and Deeming, 1982). Molenaar et al. (2010) reported that chicks hatched from water-stressed embryos were smaller and lost more than 20% of their initial mass when compared with control chicks. Layer breeders were reported to have stronger eggshells than broiler breeders (Johansson et al., 1996), which may have contributed to the increased mortality observed in layers embryos compared to broilers at the end of incubation (Everaert et al., 2008). This difference between broilers and layer strains in eggshell thickness may affect both external pipping and also negatively influence pulmonary respiration by decreasing the relative weights of the lung (Molenaar et al., 2010).

Acellular structures like the egg yolk, vitelline membrane, egg white, and eggshell not only provide nutrients but also protect the chicken embryo against physical shock and microbial infection during development (Rehault-Godbert et al., 2011). Immune function is an important physiological process that can be compromised by any reduction in metabolic resources (Moeller et al., 2013). The production, maintenance, regulation, and physiological importance of the osmotic environment in and around the avian embryo have been well described (Romanoff, 1929; Romanoff and Romanoff, 1949; Adolph, 1967). However, there is not much information available to describe the role of the osmotic environment on immune cells during the latter part of incubation. The thymus and spleen have significantly increased osmolality than serum, suggesting that lymphocytes are exposed to a certain degree of physiologic osmotic stress (Go et al., 2004). Since lymphocytes have higher osmolality, it is not clear if immune system development would be affected by an increase in water loss during incubation. Several studies have investigated the effects of moisture loss on hatchability and embryonic development, but these studies, in general, did not differentiate between the effects of moisture loss or temperature on embryonic metabolism. Hence, we hypothesized that moisture loss during embryogenesis would negatively influence immune function. The objective of this study was to investigate the effects of induced moisture loss without changes in incubator temperature on embryonic development and the immune response following an inflammatory challenge immediately post-hatch.

## Materials and Methods

The Institutional Animal Care and Use Committee at The Ohio State University approved all animal protocols (IACUC # 20140090).

### Experiment I

This experiment studied the effect of induced moisture loss in broiler and layer strain eggs on embryos weight, dry weight, and proliferation efficiency of T-lymphocytes, CD4^+^, CD8^+^, and CD4^+^CD25^+^ T-regulatory cells at ED18. Fertile leghorn eggs (*n* = 100) and commercial broiler eggs (*n* = 300; Orrville Chick hatchery, Orrville, OH, United States) were stored at 10°C and 65% relative humidity (RH) until use. All eggs were warmed to room temperature (20°C) for 12 h immediately prior to incubation. The layer and broiler eggs were stratified by weight to uniformly distribute egg weights across all the treatments prior to incubation. The eggs were set at 37.5°C and 65% RH in a single-stage incubator (Natureform, Jacksonville, FL, United States) at the OARDC Poultry Research Farm. Incubation temperature was regulated by thermistors connected to microprocessors with a temperature sensitivity of ±0.05°C. Humidity was controlled using humidity sensors. Infrared thermometers were placed in each incubator tray to monitor incubation temperature. Humidity levels and incubation temperature were logged daily.

#### Induced Water Loss

At ED8, all eggs were candled, and infertile eggs were removed. Fertile eggs were evenly distributed among six egg trays. Increased moisture loss in one-half of the Leghorn and broiler eggs was induced by drilling two, 1.5 mm diameter holes approximately 1 cm apart into the airspace with a 16G needle (BD Biosciences, Franklin Lakes, NJ, United States). The egg surface was cleaned with 70% ethanol before the holes were drilled. The Control eggs had 0 holes. Trays with experimental eggs were randomly distributed within the setter and set at 37.5°C with 65% RH for the remainder of the incubation period.

#### Moisture Loss

On ED18, moisture loss was calculated by individually weighing leghorn and broiler eggs. Embryos were sacrificed at ED18 and dry mass was obtained by drying the yolk-free embryos (*n* = 25) in an oven at 60°C until there were no further decreases in weight. Spleen, and cecal tonsils from three embryos were pooled into one replicate sample (*n* = 6) in RNAlater^®^ (Sigma-Aldrich, St. Louis, MO, United States) and stored at –70°C until further analysis. The trial was terminated at ED18.

#### CD4^+^, CD8^+^, and CD4^+^CD25^+^ T Regulatory Cell Percentage in the Spleen and Thymus

At ED18, the effect of moisture loss on CD4^+^, CD8^+^, and CD4^+^CD25^+^ T-regulatory cells was determined by flow cytometry as described previously (Shanmugasundaram et al., 2015). The splenocytes and thymocytes were collected from 18 embryos per treatment group, with three individual samples pooled into one (*n* = 6). Single-cell suspensions from the spleen and thymus pools were enriched for lymphocytes by density centrifugation over Histopaque (1.077 g/ml, Sigma-Aldrich, St. Louis, MO, United States) for 15 min at 400 *g*. The cells were incubated with a 1:250 dilution of fluorescent-isothiocyanate conjugated mouse anti-chicken CD4^+^ (Southern Biotech, Birmingham, AL, United States); 1:450 dilution of phycoerythrin-conjugated mouse anti-chicken CD8^+^ (Southern Biotech, Birmingham, AL, United States), and 1:200 dilution of unlabeled mouse IgG for 15 min. The unbound antibodies were removed by centrifugation, and the percentages of CD4^+^ and CD8^+^ cells were analyzed using a flow cytometer (Guava EasyCyte, Millipore, Taunton, MA, United sates). For the T-regulatory cell percentages, cells (1 × 10^6^) were incubated with 10 μg/ml of primary fluorescent linked mouse anti-chicken CD25^+^, 1:250 dilution of fluorescent-isothiocyanate conjugated mouse anti-chicken CD4^+^ (Southern Biotechnology Associates, Birmingham, AL, United States), and 1:200 dilution of unlabeled mouse IgG for 45 min. The unbound primary antibodies were removed by centrifugation. The percentage of CD4^+^CD25^+^ cells in the different organs was analyzed in a flow cytometer (Guava EasyCyte, Millipore, Taunton, MA, United sates) and expressed as a percentage of total CD4^+^ cells.

#### Thymocyte Proliferation Efficiency

At ED18, the effect of moisture loss on the proliferation efficiency of thymocytes was measured using the 3-[4,5-dimethylthiazol-2-yl]-2,5-diphenyl tetrazolium bromide (MTT) colorimetric assay as described previously (Shanmugasundaram et al., 2019). Thymocytes were collected from 18 embryos per treatment group, with three samples pooled into one (*n* = 6). Thymocytes were collected by density centrifugation using Histopaque (1.077 g/ml, Sigma-Aldrich, St. Luis, MO, United States). Live cells were counted by trypan blue exclusion. Thymocytes (1 × 10^4^ cells) were cultured in 200 μl of RPMI- 1640 media supplemented with 10% chicken serum and 1% penicillin, streptomycin. Cells were stimulated with 200 ng/ml phorbol 12-myristate 13-acetate (PMI), plus 50 ng/ml ionomycin (IM), in 96-well round-bottom plates for 72 h in a 5% CO_2_ incubator at 37°C. Each sample was replicated three times. At 72 h of cell culture, 20 μl of 5 mg/ml MTT (Sigma Aldrich, St. Louis, MO, United States) solution was added to the cell culture and incubated for an additional 4 h. The supernatant was removed after centrifugation. The cells were suspended in 200 μl of isopropanol plus 10% dimethyl sulfoxide and 0.04N HCl for 1 h at room temperature. The concentration of MTT formazan formed in the 96-well plates was measured on an Epoch microplate spectrophotometer at 570 nm (BIOTek, VT, United States) and reported as the mean optical density. The thymocytes proliferation percentage was calculated as follows:


Thymocyteproliferationpercentage=Mean⁢OD⁢value⁢of⁢ 2⁢-⁢hole⁢treatmentmean⁢OD⁢value⁢of⁢ 0⁢-⁢hole⁢treatmentmean⁢OD⁢value⁢of⁢ 0⁢-⁢hole⁢treatment×100

### Experiment II

This experiment studied the effect of induced moisture loss on the post-hatch immune response following a lipopolysaccharide (LPS) inflammatory challenge. The determined metrics included; body weight, splenic CD8^+^: CD4^+^ cell ratios, lymphocytes proliferation efficiency, the pro-inflammatory cytokine Interleukin (IL)1-β, IL-6, anti-inflammatory cytokine IL -10, and toll-like receptor-4 (TLR-4). The same incubation protocol described for Experiment I was subsequently repeated with 300 fertile broiler eggs (Orrville Hatchery, Orrville, OH, United States). On ED18, moisture loss was calculated as described in Experiment I and the eggs from each treatment were randomly distributed to six hatch baskets per treatment (*n* = 6), transferred to a hatcher, and incubated at 37.0°C and 70% relative humidity. Non-hatched eggs were opened at ED21, and the number of infertile eggs or dead embryos was determined. At the time of the hatch, chicks from each hatch basket were weighed individually.

#### Lipopolysaccharide Injection

On the day of hatch, chicks were randomly distributed among one of four treatments in a 2 (0 and 2-holes) × 2 (0 and 500 μg LPS) factorial setup. On d1 post-hatch, chicks in the LPS groups were injected intraperitoneally with 500 μg/kg BW LPS from *Salmonella enteritidis* (Sigma Aldrich, St. Louis, MO, United States) in 250 μl of PBS. Each treatment was replicated in six battery cages (*n* = 6) with ten chicks per replication. The chicks were housed in environmentally controlled rooms and had *ad libitum* access to water and a starter diet.

#### CD4^+^, CD8^+^, and Tregs Percentage in Thymus and Spleen

At 24 and 48 h post-LPS challenge, the effect of moisture loss on CD4^+^, CD8^+^, and CD4^+^CD25^+^ T-regulatory cells were determined by flow cytometry as described above in Experiment I.

#### Splenic and Cecal Tonsils IL1-β, IL-6, IL-10, TLR-4 and Jejunal Muc-2 Cytokine mRNA Transcription

At 24 and 48 h post-LPS injection, thymus, spleen, ∼1 cm jejunal tissue sampled proximal to Meckel’s diverticulum, and cecal tonsils were collected from each treatment group in RNAlater^®^ (Sigma-Aldrich, St. Louis, MO, United States). Samples from three chicks were pooled into one replicate sample and stored at –70°C until further analysis.

Total RNA was extracted from all experimental groups using the TRI reagent (Molecular Research Center, Cincinnati, OH, United States) following the manufacturer’s instructions. RNA concentration and purity were determined by NanoDrop (Thermo Fisher Scientific, Waltham, MA, United States) using the 260/280 and 260/230 ratios. Two μg RNA was reverse transcribed into cDNA and analyzed for IL-1β, IL-6, IL-10, and TLR-4 expression by real-time PCR (iCycler, Bio-Rad, Hercules, CA, United States) using SYBR Green. Each well contained 10 μl SYBR Green PCR master mix, 7 μl RNAse-free water using IQ5 Cycler (iCycler, Bio-Rad, Hercules, CA, United States), 2 μl (∼600 ng/μl) cDNA, 0.5 μl forward primer (5 μM), and 0.5 μl reverse primer (5 μM). To perform real-time PCR, the following machine settings were used for all genes: an initial denaturation of 95°C for 10 min (1 cycle), followed by 95°C for 15 s, and 60°C for 45 s (40 cycles). The melting profile was determined by heating samples at 65°C for 30 s and then increasing the temperature at a linear rate of 10°C/s to 95°C while continuously monitoring fluorescence. The spleen mRNA analyzed for IL-1β, IL-6, IL-10, and TLR-4 were normalized with β-actin (primer sequences and annealing temperature found on Table 1 (Shanmugasundaram and Selvaraj, 2011). The cecal tonsils mRNA analyzed for IL-1β, IL-6, IL-10, and TLR-4 were normalized with Ribosomal protein S13 (RPS13). The jejunal mRNA analyzed for Muc-2 was normalized with RPS13. The 2^–ΔΔCt^ method previously described (Livak and Schmittgen, 2001) using the Ct as the threshold cycle to calculate mRNA expression and the fold change calculated as 2^(Ct Sample^ ^–housekeeping)^/2 ^(Ct Reference^ ^–housekeeping)^. The reference group was the non-challenged 0-hole control group.

**TABLE 1:** Primer sequences for genes under study.

Gene	Primer Sequence^1^ (5′- 3′)	Annealing Temp (°C)	Product size (bp)	GenBank accession no.
IL10	F-CATGCTGCTGGGCCTGAA	57.5	94	AJ621254.1
	R-CGTCTCCTTGATCTGCTTGATG			
IL1-β	F-TCCTCCAGCCAGAAAGTGA	57.5	225	NM_204524.2
	R-CAGGCGGTAGAAGATGAAGC			
IL-6	F-ACAGCACAAAGCACCTGGCG	55	100	NM_204628.2
	R-TTGGCGAGGAGGGATTTCTGGG			
MUC-2	F-ACCAAGCAGAAAAGCTGGAA	61	257	XM_040673077.1
	R-CCTCCAGCCACCCAGTATAA			
TLR-4	F-ACCTACCCATCGGACACTTG	60	109	KP410249.1
	R-TGCCTGAGAGAGGTCAGGTT			
β-actin	F-ACCGGACTGTTACCAACACC	56	154	NM_205518.1
	R-GACTGCTGCTGACACCTTCA			
RPS13	F-CAAGAAGGCTGTTGCTGTTCG	55	169	NM_001001783.1
	R-GGCAGAAGCTGTCGATGATT			

*^1^F, forward; R, reverse.*

### Statistical Analysis

In Experiment I, data collected from multiple time points from the same eggs were analyzed using a paired student’s *t*-test to determine the effects of 0- or 2- hole on moisture loss and a student’s *t*-test to determine the effects of 0- or 2- hole on immune parameters. In Experiment II, a two-way ANOVA was performed using the fit model procedure (JMP, SAS Institute, Cary, NC, United States) to determine the effects of holes and LPS injection on measured parameters. Values reported are least-squares means ± SEM. Significance was established at *P* ≤ 0.05 and Tukey’s test was used to separate the means.

## Results

### Experiment I

#### Moisture Loss at ED18

At ED18, the layer and broiler eggs in the 2-holes treatment had a significant (*P* < 0.01) increase in moisture loss compared to the control treatments, respectively (Table 2). At ED18, there were no significant treatment differences in yolk-free embryo dry weight in either the layer or broiler embryos (Table 2).

**TABLE 2:** Effects of 2-holes drilled on the eggshell into the airspace at 8 days of incubation on moisture loss.

Treatment	Egg weight (g)			Moisture Loss (%) Dry mass (g)			
	D0	D8	D18	D 0–8	D 8–18	D 0–18	D 18
**Layers**							
0-hole	61.7	59.1	56.7	4.2^b^	4.0^b^	8.2^b^	4.4
2-holes	61.6	58.4	55.4	5.01^a^	5.01^a^	10.1^a^	3.9
*P-value*	0.91	0.65	0.4	0.01	0.01	0.01	0.22
SEM	0.6	0.6	0.6	0.1	0.1	0.1	0.3
**Broilers**							
0-hole	63	60.3	57.8	4.3	4.1^b^	8.4^b^	5.3
2-holes	63.3	60.5	57.2	4.4	5.05^a^	9.9^a^	4.8
*P-value*	0.58	0.67	0.27	0.43	0.001	0.001	0.79
SEM	0.2	0.3	0.4	0.1	0.1	0.1	0.1

*Fertile layer eggs (n = 100) and broiler eggs (n = 300) were incubated at standard (37.5°C) from embryonic day (ED) 1–18. At ED8, half the eggs were incubated at 37.5°C, 2-holes were drilled on the eggshell into the airspace, and remained eggs served as control groups. The eggs were continued to be incubated at 37.5°C until ED18. Egg weights were measured before set in the incubator, D8, and D18. Moisture loss was calculated as a percentage from D8, D8–18, and D0–18. Dry mass of yolk-free embryos weight was calculated by drying the embryos in an oven at 60°C until there were no further decreases in weight. Means with no common superscripts differ significantly (P ≤ 0.05). n = 6.*

#### Thymocytes Proliferation

**FIGURE 1:**
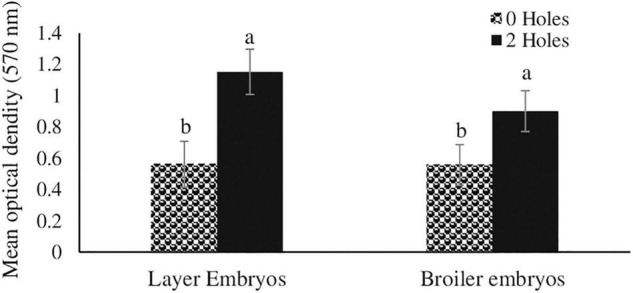
Thymocyte proliferation efficiency in layer and broiler embryos at ED18 (Experiment I). Fertile layer and broiler eggs were incubated at standard (37.5°C) incubation temperature from embryonic day (ED) 1–8. At ED8, the eggs incubated at 37.5°C were drilled either 0-hole or 2-holes in the eggshell above the air cell and continued to incubate until ED18. At ED18, 1 × 10^5^ thymocytes were stimulated *in vitro* with 200 ng/ml phorbol 12-myristate 13-acetate (PMI), plus 50 ng/ml ionomycin (IM). At 72 h of culture, thymocyte proliferation was analyzed by MTT assay. Means with no common superscripts differ significantly (*P* ≤ 0.05; *n* = 6).

In Experiments I, inducing moisture loss had a significant effect on *in vitro* thymocyte proliferation. Thymocytes from both the layer (104%) and broiler (62%) embryos in the 2-holes treatment had significantly increased *in vitro* proliferation compared with the control embryos (*P* ≤ 0.05; Figure 1).

#### CD8^+^/CD4^+^ Ratio and CD4^+^CD25^+^ Cell Percentages in Thymus and Spleen at ED18

At ED18, layer and broiler embryos in the 2-holes treatment had an approximate twofold increase in the splenic CD8^+^/CD4^+^ ratio compared to the control treatment (*P* ≤ 0.05; Table 3). The layer embryos from the 2-holes treatment had a significant increase in CD4^+^CD25^+^ cell percentages in both the thymus and spleen whereas the broiler embryos from 2-holes treatment only had a significant increase in CD4^+^CD25^+^ cell percentage in the spleen (*P* ≤ 0.05; Table 3).

**TABLE 3:** CD8^+^/CD4^+^ ratio and CD4^+^CD25^+^ cell percentages at ED18 in thymus and spleen of layer and broiler embryos (Experiment I).

		Treatment	Thymus		Spleen	
ED18		Holes	CD8^+^/CD4^+^	% Tregs	CD8^+^/CD4^+^	% Tregs
**Layer embryos**		0	1.4	2.4^b^	5.2^b^	3.5^b^
		2	2.5	4.5^a^	10.1^a^	6.5^a^
	*P-value*		0.09	0.02	0.01	0.01
	SEM		0.2	0.5	0.6	0.5
**Broiler embryos**		0	1.1	2.2	6.6^b^	5.5^b^
		2	2.3	3.4	9.6^a^	8.1^a^
	*P-value*		0.14	0.07	0.05	0.01
	SEM		0.2	0.3	0.6	0.5

*Fertile broiler eggs were incubated at standard (37.5°C) incubation temperature from embryonic day (ED) 1–8. At ED8, the eggs incubated at 37.5°C were drilled either 0-hole or 2-holes in the eggshell above the air cell and continued to incubate until ED18. The embryos from each treatment group were transferred to the hatcher and maintained at 37.0°C and 70% relative humidity until hatch. At ED18 thymus, and spleen were collected from all the treatment groups and analyzed for CD8^+^_/_CD4^+^ ratio and CD4^+^/CD25^+^ by flow cytometry after staining with fluorescent-linked anti-chicken CD4, CD8, and CD25 antibodies. Means with no common superscripts differ significantly (P ≤ 0.05). n = 6.*

#### Splenic and Cecal Tonsil IL1-β, IL6, IL-10, and TLR-4 mRNA Transcription at ED18

At ED18, layer and broiler embryos from 2-holes treatment both had a significant increase in splenic IL1-β, IL-6, IL-10, and TLR-4 mRNA transcription compared to that in the control group (*P* ≤ 0.05; Table 4). Layer and broiler embryos in the 2-holes treatment had a 2.9 to 8.2 fold increase in transcription of the different splenic cytokine mRNAs studied compared to the control group. At ED18, layer and broiler embryos from the 2-holes treatment had a significant increase in cecal tonsil IL-6, IL-10, and TLR-4 mRNA transcription compared to the control group (*P* ≤ 0.05).

**TABLE 4:** IL1-β, IL6, IL10, and TLR-4 mRNA expression at ED18 in spleen and cecal tonsils of layer and broiler embryos (Experiment I).

	Spleen	0-hole-Control	2-holes-Control	*P-value*	SEM
**Layer Embryos**					
	IL1-β	1.0^b^	4.6^a^	0.01	0.5
	IL-6	1.0^b^	8.2^a^	0.01	0.3
	IL10	1.0 ^b^	5.1^a^	0.01	0.2
	TLR-4	1.0 ^b^	4.1^a^	0.01	0.3
**Broiler Embryos**					
	IL1-β	1.0 ^b^	2.9^a^	0.01	0.1
	IL-6	1.0 ^b^	4.3^a^	0.01	0.3
	IL10	1.0 ^b^	5.6^a^	0.01	0.4
	TLR-4	1.0 ^b^	4.6^a^	0.01	0.3
	**Cecal tonsils**				
**Layer Embryos**					
	IL1-β	1.0	2.6	0.07	0.2
	IL-6	1.0^b^	14.5^a^	0.01	0.5
	IL10	1.0^b^	5.3^a^	0.01	0.2
	TLR-4	1.0^b^	3.1^a^	0.01	0.3
**Broiler Embryos**					
	IL1-β	1.0	2.5	0.2	0.4
	IL-6	1.0^b^	4.6^a^	0.01	0.3
	IL10	1.0^b^	3.2^a^	0.01	0.2
	TLR-4	1.0^b^	3.8^a^	0.01	0.2

*Fertile broiler eggs were incubated at standard (37.5°C) incubation temperature from embryonic day (ED) 1–8. At ED8, the eggs incubated at 37.5°C were drilled either 0-hole or 2-holes in the eggshell above the air cell and continued to incubate until ED18. At ED18, spleen and cecal tonsils from both layer and broiler embryos were collected and analyzed for mRNA by real-time PCR analysis. The mRNA content of the spleen was normalized with β-Actin mRNA content, and the mRNA content of the cecal tonsil was normalized with RPS13 mRNA content. The fold change was calculated with the 0-hole control groups. Means with no common superscripts differ significantly (P ≤ 0.05). n = 6.*

### Experiment II

#### Broiler Eggs Hatchability Percentage and Hatch Weight

In Experiment II, hatchability in the 0-hole and the 2-holes treatment was 88.9 and 74%, respectively. The average hatch weight of broiler chicks in the 2-holes treatment group was 42.6 g, while that in the control groups was 43.4 g. The chicks hatched from 2-holes treatment had a 1.9% decrease in body weight at the time of hatch than that in the control group.

#### CD8^+^/CD4^+^ Ratio and CD4^+^CD25^+^ Cell Percentages in Thymus and Spleen

There were significant interaction effects between hole number and LPS on the CD8^+^/CD4^+^ ratio at 24 h post-LPS challenge in both the thymus and spleen (*P* ≤ 0.05; Table 5). Chicks hatched from the 2-holes treatment and challenged with LPS had an approximately two fold higher CD8^+^/CD4^+^ ratio compared to that in both the control group and 2-holes treatment with 0 μg LPS. Birds hatched from the 0-hole treatment and challenged with LPS had a significantly higher CD8^+^/CD4^+^ ratio compared to the control group.

**TABLE 5:** CD8^+^/CD4^+^ ratio and CD4^+^CD25^+^ cell percentages at 24 and 48 h post-LPS injection in thymus and spleen (Experiment II).

	0-hole-Control	2-holes-Control	0-hole-LPS challenge	2-holes-LPS challenge	Main effects		Interaction	SEM
					*Hole P-value*	LPS challenge *P-value*	Hole × LPS challenge *P-value*	
**24 h**								
**Thymus**								
CD8/CD4	2.5^c^	2.8^c^	4.0^b^	5.7^a^	0.01	0.04	0.01	0.2
Tregs	0.5^c^	1.6^b^	2.0^b^	3.6^a^	0.05	0.01	0.01	0.2
**Spleen**								
CD8/CD4	1.1^c^	0.9^c^	2.2^b^	3.1^a^	0.13	0.05	0.04	0.3
Tregs	3.1^b^	3.5^b^	4.0^b^	5.0^a^	0.26	0.07	0.05	0.3
**48 h**								
**Thymus**								
CD8/CD4	2.8	2.0	4.6	4.4	0.18	0.03	0.32	0.1
Tregs	2.2^b^	2.8^b^	3.7^b^	5.8^a^	0.34	0.05	0.05	0.1
**Spleen**								
CD8/CD4	1.6^b^	1.6^b^	4.1^a^	2.5^b^	0.48	0.05	0.05	0.3
Tregs	2.3^c^	3.8^bc^	4.3^ab^	5.9^a^	0.05	0.07	0.05	0.4

*Fertile broiler eggs were incubated at standard (37.5°C) incubation temperature from embryonic day (ED) 1–8. At ED8, the eggs incubated at 37.5°C were drilled either 0-hole or 2-holes in the eggshell above the air cell and continued to incubate until ED18. The embryos from each treatment group were transferred to the hatcher and maintained at 37.0°C and 70% relative humidity until hatch. At D1, hatchlings from each treatment group were challenged intraperitoneally with either 0 or 500 μg lipopolysaccharide (LPS)/kg BW in a 2 (0-hole, 2-holes) × 2 (control, LPS challenge) factorial design. At 24 and 48 h post-LPS injection, thymus, and spleen from all the treatment groups were collected and analyzed for CD4^+^_/_CD8^+^ ratio and CD4^+^/CD25^+^by flow cytometry after staining with fluorescent-linked anti-chicken CD4, CD8, and CD25 antibodies. Means with no common superscripts differ significantly (P ≤ 0.05). n = 6.*

There was also a significant interaction effect between hole number and LPS on the CD8^+^/CD4^+^ ratio at 48 h post-LPS challenge in the spleen (*P* ≤ 0.05; Table 4). At 48 h post-LPS challenge, chicks hatched from the 0-hole treatment and challenged with LPS had a significantly higher splenic CD8^+^/CD4^+^ ratio than that in the control group, while birds hatched from 2-holes treatment and challenged with LPS had a comparable CD8^+^/CD4^+^ ratio to that in the control group (*P* = 0.05).

There was no significant interaction effect between hole and LPS on the thymic CD8^+^/CD4^+^ ratio. However, there was a significant main effect of LPS challenge on the thymic CD8^+^/CD4^+^ ratio at 48 h post-LPS challenge (*P* < 0.05). Birds injected with LPS had a significantly higher thymic CD8^+^/CD4^+^ ratio compared to the 0 μg LPS group.

There were significant interactions (*P* ≤ 0.05) between the hole number and LPS challenge on CD4^+^CD25^+^ cell percentages in the thymus and spleen at both 24 and 48 h post-LPS challenge. Chicks hatched from 2-holes treatment and challenged with LPS had an approximately 1.5-fold increase in CD4^+^CD25^+^ percentage compared to that in the control group.

#### Thymocytes Proliferation at 24 and 48 h Post-lipopolysaccharide Injection

**FIGURE 2:**
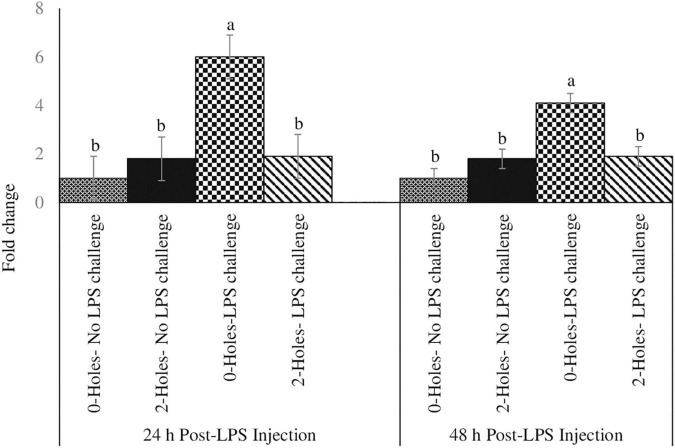
Thymocyte proliferation efficiency at 24 and 48 h post-LPS injection (Experiment II). Fertile broiler eggs were incubated at standard (37.5°C) incubation temperature from embryonic day (ED) 1–8. At ED8, the eggs incubated at 37.5°C were drilled either 0-hole or 2-holes in the eggshell above the air cell and continued to incubate until ED18. The embryos from each treatment group were transferred to the hatcher and maintained at 37.0°C and 70% relative humidity until hatch. At D1, hatchlings from each treatment group were challenged intraperitoneally with either 0 or 500 μg lipopolysaccharide (LPS)/kg BW in a 2 (0-hole, 2-holes) × 2 (control, LPS challenge) factorial design. At 24 and 48 h post-LPS injection, 1 × 10^5^ thymocytes were stimulated *in vitro* with 200 ng/ml phorbol 12-myristate 13-acetate (PMI), plus 50 ng/ml ionomycin (IM). At 72 h of culture, thymocyte proliferation was analyzed by MTT assay. Means with no common superscripts differ significantly (*P* ≤ 0.05; *n* = 6). *P*-values: 24 h post- LPS challenge; holes × LPS challenge *P* = 0.02; Holes *P* = 0.01, LPS challenge *P* = 0.05. 48 h post- LPS challenge; holes × LPS challenge *P* = 0.03; Holes *P* = 0.01, LPS challenge *P* = 0.05.

In broiler chicks, there was a significant interaction between 0 and 2-holes treatments and LPS on thymocyte proliferation at 24 and 48 h (*P* < 0.05) post-LPS challenge. At 24 and 48 h post-hatch, chicks hatched from 2-holes eggs and challenged with LPS had a significant increase in thymocyte proliferation (*P* ≤ 0.05; Figure 2) at 24 h (42%) and 48 h (37%) when compared with control chicks (0-hole; 0 μg LPS), respectively.

#### Splenic IL1-β, IL-6, IL-10, and TLR-4 mRNA Transcription at 24 and 48 h Post-lipopolysaccharide Injection

At 24 h post-LPS challenge, there were significant interaction effects between the hole and LPS challenge on IL1-β and TLR-4 mRNA transcription in the spleen (*P* < 0.05; Table 6). At both 24 and 48 h post-LPS challenge, chicks hatched from the 0-hole treatment and challenged with LPS had significantly higher splenic IL1-β and TLR-4 mRNA transcription compared to control, non-challenge group; while chicks from the 2-holes treatment and with LPS challenge had comparable IL1-β and TLR-4 mRNA transcription levels to the control group (*P* < 0.05).

**TABLE 6:** Splenic IL1-β, IL-6, IL10, and TLR-4 mRNA expression at 24 and 48 h post-LPS injection (Experiment II).

Spleen	0-hole-Control	2-holes-Control	0-hole-LPS	2-holes-LPS	Main effects		Interaction	SEM
					*Hole P-value*	LPS challenge *P-value*	Hole × LPS challenge *P-value*	
**24 h**								
IL1-β	1.0^b^	0.98^b^	7.2^a^	3.3^b^	*0.07*	0.01	0.03	0.5
IL-6	1.0	1.4	5.6	2.6	*0.37*	0.05	0.24	0.7
IL10	1.0	2.8	2.1	5.2	*0.01*	0.03	0.37	0.4
TLR-4	1.0^b^	1.7^b^	5.6^a^	1.8^b^	*0.07*	0.01	0.01	0.4
**48 h**								
IL1-β	1.0^b^	1.1^b^	3.8^a^	1.5^b^	*0.02*	0.01	0.01	0.2
IL-6	1.0	2.4	1.8	2.2	*0.16*	0.63	0.41	0.3
IL10	1.0	1.9	4.1	2.8	*0.81*	0.03	0.25	0.5
TLR-4	1.0^b^	2.1^b^	3.2^a^	1.7^b^	*0.78*	0.23	0.05	0.4

*Fertile broiler eggs were incubated at standard (37.5°C) incubation temperature from embryonic day (ED) 1–8. At ED8, the eggs incubated at 37.5°C were drilled either 0–holes or 2-holes in the eggshell above the air cell and continued to incubate until ED18. The embryos from each treatment group were transferred to the hatcher and maintained at 37.0°C and 70% relative humidity until hatch. At D1, hatchlings from each treatment group were challenged intraperitoneally with either 0 or 500 μg lipopolysaccharide (LPS)/kg BW in a 2 (0-hole, 2- holes) × 2 (control, LPS challenge) factorial design. At 24 h, 48 h post-LPS injection, spleen from all the treatment groups were collected and analyzed for mRNA by real-time PCR analysis. The mRNA content was corrected for reference gene β-Actin mRNA content and normalized to the mRNA content of the 0-hole-control groups. Means with no common superscripts differ significantly (P ≤ 0.05). n = 6.*

There was a significant main effect of LPS challenge on splenic IL-6 and IL-10 mRNA transcription at 24 h and on splenic IL-10 at 48 h post-LPS challenge (*P* < 0.05). Birds challenged with LPS had significantly higher IL-6 and IL-10 mRNA transcription compared to that in the 0 μg LPS group.

#### Cecal Tonsils IL1-β, IL-6, IL-10, and TLR-4 mRNA Transcription at 24 and 48 h Post-lipopolysaccharide Injection

There were significant interaction effects between the hole number and LPS challenge on IL1-β, IL-6, and TLR-4 mRNA transcription in the cecal tonsils at 24 h post-LPS challenge (*P* < 0.05; Table 7). At 24 h, chicks hatched from 0-hole treatment and challenged with LPS had significantly higher cecal tonsil IL1-β, IL-6, and TLR-4 mRNA transcription than control chicks, while those chicks in the 2-holes treatment and challenged with LPS had comparable IL1-β, IL-6, and TLR-4 mRNA transcription compared to the control group (*P* < 0.05).

**TABLE 7:** Cecal tonsils IL1-β, IL-6, and IL-10 mRNA expression at 24 and 48 h post-LPS injection (Experiment II).

Cecal tonsils	0-hole Control	2-holes Control	0-hole- LPS	2-holes-LPS	Main effects		Interaction	SEM
					*Hole P-value*	LPS challenge *P-value*	Hole × LPS challenge *P-value*	
**24h**								
IL1-β	1.0^b^	1.1^b^	11.8^a^	2.3^b^	*0.01*	0.01	0.01	0.6
IL6	1.0^b^	1.8^b^	3.9^a^	1.9^b^	*0.36*	0.02	0.03	0.3
IL10	1.0	1.4	2.6	4.7	*0.09*	0.01	0.26	0.4
TLR4	1.0^b^	1.6^b^	4.1^a^	2.1^b^	*0.20*	0.01	0.01	0.3
**48 h**								
IL1-β	1.0	1.1	1.0	1.5	*0.76*	0.07	0.64	0.2
IL6	1.0	1.2	2.4	1.5	*0.53*	0.17	0.28	0.2
IL10	1.0^b^	1.8^b^	5.7^a^	2.8^b^	*0.14*	0.01	0.01	0.4
TLR4	1.0	1.5	2.5	2.2	*0.83*	0.10	0.50	0.3

*Fertile broiler eggs were incubated at standard (37.5°C) incubation temperature from embryonic day (ED) 1–8. At ED8, the eggs incubated at 37.5°C were drilled either 0-hole or 2-holes in the eggshell above the air cell and continued to incubate until ED18. The embryos from each treatment group were transferred to the hatcher and maintained at 37.0°C and 70% relative humidity until hatch. At D1, hatchlings from each treatment group were challenged intraperitoneally with either 0 or 500 μg lipopolysaccharide (LPS)/kg BW in a 2 (0-hole, 2 holes) × 2 (control, LPS challenge) factorial design. At 24 and 48 h post-LPS injection, cecal tonsils from all the treatment groups were collected and mRNA was quantified by real-time PCR analysis. The mRNA content was corrected for reference gene RPS13 mRNA content and normalized to the mRNA content of the 0-hole-control group. Means with no common superscripts differ significantly (P ≤ 0.05). n = 6.*

There was a significant main effect of LPS challenge on IL-10 mRNA transcription at 24 h. Chicks challenged with LPS had significantly higher IL-10 mRNA transcription when compared with 0 μg LPS group.

At 48 h post-LPS challenge, there were significant interaction effects between hole number and LPS challenge on IL-10 mRNA transcription in the cecal tonsils (*P* < 0.05). Chicks hatched from 0-hole treatment and challenged with LPS had significantly higher cecal tonsil IL-10 mRNA transcription than that in the control chicks, while challenged chicks from the 2-holes treatment and had comparable IL-10 mRNA transcription to the control group (*P* = 0.05).

#### Jejunal Muc-2 Gene Expression at 24 and 48 h Post-lipopolysaccharide Challenge

**FIGURE 3:**
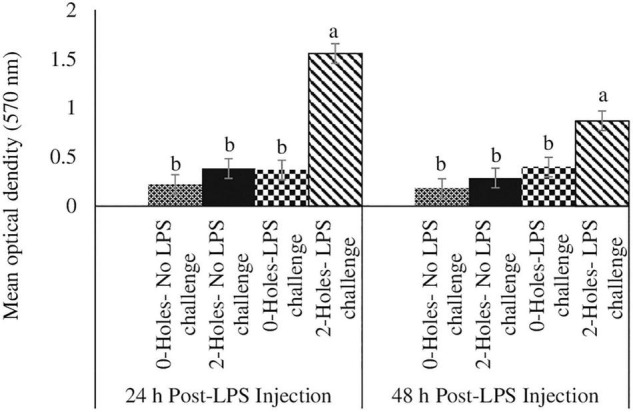
Jejunal Muc2 gene expression at 24 and 48 h post-LPS injection (Experiment II). Fertile broiler eggs were incubated at standard (37.5°C) incubation temperature from embryonic day (ED) 1–8. At ED8, the eggs incubated at 37.5°C were drilled either 0-hole or 2-holes in the eggshell above the air cell and continued to incubate until ED18. The embryos from each treatment group were transferred to the hatcher and maintained at 37.0°C and 70% relative humidity until hatch. At D1, hatchlings from each treatment group were challenged intraperitoneally with either 0 or 500 μg lipopolysaccharide (LPS)/kg BW in a 2 (0-hole, 2-holes) × 2 (control, LPS challenge) factorial design. At 24 and 48 h post-LPS injection, jejunal tissue from all the treatment groups was collected and analyzed for MUC2 mRNA by real-time PCR analysis. The mRNA content was normalized to the mRNA content of housekeeping gene RPS13 and data presented as a fold change compared to the mRNA content of the no hole -control group. Means with no common superscripts differ significantly (*P* ≤ 0.05). *n* = 6. *P*-values: 24 h post- LPS challenge; holes × LPS challenge *P* = 0.02; Holes *P* = 0.11, LPS challenge *P* = 0.11. 48 h post- LPS challenge; holes × LPS challenge *P* = 0.01; Holes *P* = 0.03, LPS challenge *P* = 0.01.

There were significant interaction effects between the hole number and LPS challenge on Muc-2 mRNA transcription in the jejunum (*P* < 0.05; Figure 3). At 24 and 48 h post-LPS challenge, chicks hatched from 0-hole treatment and challenged with LPS had significantly higher Muc-2 mRNA transcription than that in the control group, while chicks from the 2-holes treatment and challenged with LPS had comparable Muc-2 mRNA transcription compared with control chicks.

## Discussion

Osmoregulation is the maintenance of the proper concentrations of water and solutes. Any changes in the appropriate ratios are generally balanced by an equal and opposite loss or gain of water (Molnar and Gair, 2013). In biological systems, fluid compartments between and within the cells are separated by semipermeable membranes, and the ability to regulate the intracellular and extracellular solute microenvironments is critical in maintaining cellular homeostasis (Shoval and Alon, 2010). The developing avian embryo contains rudimentary tissues to maintain osmoregulation and relatively very little is known about the consequences of excess water loss on osmoregulatory mechanisms and the development of the avian embryonic immune system. Hence, the goal of this study was to identify the effect of an induced moisture loss on selected immune responses of broiler and layer embryos at ED18 and the acute inflammatory response in broiler chicks to an LPS challenge on the day of hatch.

In Experiment I, the average weight of eggs from a layer strain was 61 g while the broiler hatching eggs’ average was 63 g. The eggs in both treatment groups lost 10% moisture at ED18. The loss of water occurs via diffusion through pores in the shell and, is an important regulatory process during incubation (Paganelli, 1980). Moisture loss includes both the water content of the egg along with metabolic water produced from the oxidation of lipids, particularly during the later stages of embryonic development (Ar and Rahn, 1980). At ED18, moisture loss was approximately 2% greater in the 2-holes treatment (2-holes, 10.1%; 0-hole, 8.20%; *P* < 0.01, Table 2) in layer embryos; and 1.5% (2-holes, 9.9%; 0-hole, 8.4%; *P* < 0.01) in broiler embryos. Similar results were previously observed (Davis et al., 1988; Buhr, 1995) for avian embryos incubated at 69% RH (7.6% moisture loss). In chicken eggs, hatching success is normally achieved if eggs lose approximately 7 g of water, which would represent 12% for 60 g of the initial egg mass. A major increase or decrease in total water loss during the 21-day incubation period may decrease hatching success (Barott, 1937; Landauer, 1967; Lundy, 1969). Commercial hatcheries maintain RH at 55% during the first 15 days of incubation with an increase to 60% RH after 15 days to maintain optimal hatchability (Cartwright, 2000). An increase in RH will decrease the moisture loss from the egg whereas a decrease in RH will increase water evaporation from the egg (Davis et al., 1988; Hamdy et al., 1991). In this study, the eggs were incubated at relatively higher RH (65%) with a constant incubator set temperature (37.5°C) throughout the experiment. When eggs were incubated at a constant temperature, decreasing humidity caused increased the incubation time and decreased the chick weight (Romanoff, 1929; Townsley, 1931). Eggs were incubated at a higher RH of 65% to minimize excessive water loss and subsequent embryonic mortality (Bruzual et al., 2000). The small increase in the moisture loss in 2-holes treatment could also be a result of water recycling via osmoregulation in the chorioallantoic membrane to minimize dehydration of embryos (Hoyt, 1979; Simkiss, 1980). This moisture loss did not affect the dry mass of the yolk-free embryos suggesting that induced moisture loss occurred in extra-embryonic membranes, but not from embryonic body tissues (Van der Pol et al., 2013). The increased moisture loss observed in this study at ED18 decreased the hatchability percentage and this is consistent with the earlier studies (Romanoff, 1929; Davis et al., 1988).

Though previous studies have examined the effect of dehydration on different physiological systems during incubation (Davis et al., 1988), not much information is available on the effects of embryonic water loss and the development of the avian embryonic immune system. To the best of our knowledge, we are the first group to demonstrate that induced moisture loss altered the immune indices of the embryos. In Experiment I, thymocytes, stimulated with PMA + Ionomycin, had increased proliferation in the 2-holes treatment. The chorioallantoic membrane is associated with the active transport of Na + ions from the allantoic fluid into the blood, calcium transport from the eggshell (Stewart and Terepka, 1969; Hoyt, 1979; Graves et al., 1986; Gabrielli and Accili, 2010). The increase in moisture loss likely induced a hyperosmotic environment and altered the Na^+^, K^+^, and Ca^2+^ ion transporter channels in T cells and subsequently stimulated T cell receptors to increase thymocyte proliferation (Feske et al., 2012). The induced moisture loss during embryogenesis could have activated the immune response thereby reducing BW by the time of hatch (1.9%), suggesting a physiologic trade-off between immune response versus BW.

The induced moisture loss also significantly increased the percentage of T-regulatory cells in the thymus and spleen and the splenic CD4^+^/CD8^+^ ratio at ED 18 compared with the control treatment. The activation and clonal expansion of CD4^+^ T cells is a tightly regulated process that stimulates the proliferation of resting CD4^+^ T cells and is critical for the activation of immune responses (Li et al., 2014). The CD4^+^CD25^+^ T regulatory cells (Tregs) are a unique subset of T-cells that regulate immune response and establish peripheral tolerance. The data in Experiment I suggested that induced moisture loss likely induced some degree of osmotic stress. Induced water losses ≥ 20% depletes allantoic fluid as resulted in prolonged osmotic stress in chicken embryos (Davis et al., 1988). Osmotic stress has been reported to alter the homeostasis of T cells (Brocker et al., 2012). At ED18, induced moisture loss increased the splenic Tregs percentage and upregulated IL-10 mRNA transcription in spleen and cecal tonsils, suggesting that the spleen and cecal tonsils were sensitive to the induced osmotic changes at ED18. Moreover, avian embryos most likely sensed the extracellular hyperosmolarity at the cell membrane (Lunn and Rozengurt, 2004) and subsequently increased the transcription of splenic IL-6, TLR-4, and IL-10 in both layers and broiler embryos at ED18. Induced moisture loss altered the function of the immune system by increasing the proliferation of T cells and modulating the production of IL1-β, IL-6, and IL-10 (Coimbra et al., 1995). These changes in cytokine expression are most likely due to the activation of the conserved adaptive mechanisms in response to osmotic imbalances during embryonic development (Hubert et al., 2004). Taken together, the observed increase in the CD8^+^/CD4^+^ ratio and Tregs in embryos with induced moisture loss is likely a compensatory mechanism to facilitate immune and inflammatory responses for possible pathogen infections.

To determine if the induced moisture loss could play a role in the protection of newly hatched chicks to an inflammatory challenge, broiler chicks, hatched from control and induced moisture loss eggs, were injected with LPS. Moisture loss during incubation is a major factor that dysregulates immune response and increases mortality post-hatch (Yassin et al., 2009). Cells have developed several adaptive response mechanisms, by secreting cytokines to counter osmotic stress and restore osmotic equilibrium (Brocker et al., 2012). Chicks hatched from eggs with 2-holes and challenged with LPS had a significant increase in thymocyte proliferation compared to the control group is consistent with Galindo-Villegas et al. (2016) who reported that newly hatched germ-free (GF) zebrafish sense the hyperosmolarity of the aquatic environment and mount a protective adaptive immune response.

Osmotic stress can also activate the intracellular MAP kinase pathway and a subsequent non-specific inflammatory response via the release of selected cytokines (Uhlik et al., 2003). Studies with osmotic stress reported that human aortic endothelial cells and peripheral blood mononuclear cells secrete IL-1β (Shapiro and Dinarello, 1997; Fernandes et al., 2009) and rat peritoneal macrophages secrete IL-6 (Wade, 2002). Exposure to osmotic stress reprograms the inflammatory responses to a subsequent LPS challenge resulting in decreased pro-inflammatory cytokines in mice (Pimentel et al., 2019). In our study, the mRNA transcription of pro-inflammatory genes IL-1β and IL-6 was reduced but the transcription of the anti-inflammatory cytokine IL-10 was not altered in the spleen at either 24 or 48 h post-LPS challenge. The data reported herein suggest that induced moisture loss during embryogenesis causes LPS tolerance via inhibition of TLR-4 signaling at 24 h post-LPS challenge. An earlier study identified similar LPS tolerance through inhibition of TLR signaling (Foster et al., 2007). Further, comparable IL-10 production in the spleen in response to the LPS challenge indicates that induced moisture loss during embryogenesis may precondition the chicks toward LPS tolerance (Melo et al., 2010; Pimentel et al., 2019).

Among mucin isoforms, Muc-2 is the predominant isoform in the chick intestine, and Muc-2 transcription can be modulated by local inflammatory activity (Oh et al., 2019). The LPS challenge in the chicks hatched from the 2-holes treatment did not influence Muc-2 mRNA transcription at either 24 and 48 h compared to the control groups. This is most likely due to the induced moisture loss was not sufficient enough to activate NF-κB which is necessary for the induction of Muc-2 expression and the subsequent modulation of the gut mucosal immune response. On the other hand, the LPS challenge did increase Muc-2 transcription in the chicks hatched from 0-hole treatment. Earlier reports identified that LPS induced overexpression of Muc-2 in biliary epithelial cells and chicken jejunum through TLR-4 signaling (Zen et al., 2002; Oh et al., 2019).

## Conclusion

The induced moisture loss was sufficient to decrease BW at hatch by 2%, increase thymocyte proliferation at ED18, 24 and 48 h post-LPS challenge, and decrease pro-inflammatory cytokines transcription without altering IL-10. Our data suggest that inducing moisture loss reprograms gene transcription in embryos to enhance cell survival via proliferation. Our study also suggests that osmoregulation is critical for regulating the immune system. Induced moisture loss could regulate specific gene patterns depending on the severity of osmotic stress. In future studies, the determination of osmoregulatory transcription factor NFAT5 transcription might help us to better understand how osmotic stress reprograms gene expression during embryogenesis.

## Data Availability Statement

The original contributions presented in the study are included in the article/supplementary material, further inquiries can be directed to the corresponding author/s.

## Ethics Statement

The animal study was reviewed and approved by the Institutional Animal Care and Use Committee at The Ohio State University approved all animal protocols.

## Author Contributions

JG: methodology, writing-review, and editing. MSL: conceptualization, funding acquisition, project administration, writing-review and editing. RS: conceptualization, data curation, formal analysis, investigation, methodology, validation, writing – original draft, writing – review and editing. All authors: contributed to the article and approved the submitted version.

## Conflict of Interest

The authors declare that the research was conducted in the absence of any commercial or financial relationships that could be construed as a potential conflict of interest.

## Publisher’s Note

All claims expressed in this article are solely those of the authors and do not necessarily represent those of their affiliated organizations, or those of the publisher, the editors and the reviewers. Any product that may be evaluated in this article, or claim that may be made by its manufacturer, is not guaranteed or endorsed by the publisher.
